# Influence of the Direction of Mixture Compaction on the Selected Properties of a Hemp-Lime Composite

**DOI:** 10.3390/ma14164629

**Published:** 2021-08-17

**Authors:** Przemysław Brzyski, Piotr Gleń, Mateusz Gładecki, Monika Rumińska, Zbigniew Suchorab, Grzegorz Łagód

**Affiliations:** 1 Faculty of Civil Engineering and Architecture, Lublin University of Technology, Nadbystrzycka 40, 20-618 Lublin, Poland; p.glen@pollub.pl (P.G.); mateusz.gladecki@pollub.edu.pl (M.G.); monika.ruminska@pollub.edu.pl (M.R.); 2 Faculty of Environmental Engineering, Lublin University of Technology, Nadbystrzycka 40B, 20-618 Lublin, Poland; z.suchorab@pollub.pl

**Keywords:** hemp-lime, shives, anisotropy, thermal conductivity, capillary uptake, mechanical properties

## Abstract

The aim of the research presented in the article was to check the differences in the hygro-thermal and mechanical properties of hemp-lime composites with different shives fractions, depending on the direction of mixture compaction. The research part of the paper presents the preparation method and investigation on the composites. Thermal conductivity, capillary uptake, as well as flexural and compressive strengths were examined. Additionally, an analysis of the temperature distribution in the external wall insulated with the tested composites was performed. The results confirm that the direction of compaction influences the individual properties of the composites in a similar way, depending on the size of the shives. The differences are more pronounced in the case of the composite containing longer fractions of shives. Both thermal conductivity of the material and the capillary uptake ability are lower in the parallel direction of the compaction process. Composites exhibit greater stiffness, but they fail faster with increasing loads when loaded in the direction perpendicular to compaction.

## 1. Introduction

Anisotropic materials have different properties, depending on the direction in which they are tested. This phenomenon is a feature of the materials that exist in a monocrystalline form, but polycrystalline bodies may also have anisotropic properties due to texture or the influence that the decomposition of impurities in their structure may have [1].

The influence of anisotropy may be important both in the mechanical tests of the material and in the tests of physical and thermal properties. In this case, it depends on the specific type or type of the material and may only affect the mechanical or physical properties of the tested samples. Depending on the materials tested, the influence of anisotropy on the values of capillary rise, compressive strength, tensile strength, and thermal conductivity was demonstrated [2,3,4,5,6]. The influence of the anisotropy phenomenon can be observed in the materials of organic origin. In the case of wood, the differences in the mechanical properties depending on the direction of load application lie in the complex structure of the material [7].

Anisotropic properties may be demonstrated by the composite materials based on fibers (of the natural or synthetic origin). Their orientation, resulting from mixing and compacting the mixture, affects the various properties of the composite, mainly the tensile strength [8,9,10,11]. Aggregates of irregular shapes also influence the properties of the composites. Their arrangement depends on the direction of mixture compaction. An example of such a material is a composite based on lime and hemp shives. Hemp-lime composite is used as the insulating filling of a timber frame structure of the walls—in monolithic form or in the form of blocks. Hemp shives constitute the aggregate used in the composite. Different sizes of shives are used, for example a thickness of 0.063–8 mm and a length of 2.33–7.42 mm [12,13]; length 0–5 mm and 0–20 mm [14]; length 2.5–5.0 mm [15], as well as 2.7 mm and 8.4 mm [16]. The size of the shives affects the properties of the composite. Fine fractions cause a greater demand for a binder, which results in increases in density, strength, and thermal conductivity [12,13,16]. Longer shives, in turn, have a positive effect on the flexural strength of the composite [16]. The finer the shives fractions, the easier and faster the hemp-lime mixture is homogenized during mixing. When compacting the mixture, the size of the shives also affects the orientation of the shives in the composite. Longer fractions tend to be perpendicular to the compaction direction [17,18].

The direction in which the mixture is compacted may change the orientation of the hemp shives, which may in turn alter the structure of the material, e.g., the size and distribution of pores. Nozahic et al. [19] proved that the direction of force application to the composite influences its strength, stiffness, and the failure model of concrete based on lime and organic aggregates. Williams et al. [20] described similar observations on the example of a lime-hemp composite. Nguyen et al. [17] and Pierre et al. [18] showed that the direction of compaction of composites also affects their thermal conductivity properties. In turn, the conducted studies show that the shape and orientation of the pores affect the thermal and mechanical properties of porous building materials [21,22].

The transport of water and the heat flux through the material depend, among others, on the distribution of its pores and the direction of the capillary pores. In the case of fibrous materials, the path of the pores will depend on the direction of the fibers [23].

The article presents research on the hemp-lime composites prepared according to two recipes differing in the type (size) of hemp shives. The difference in the basic properties of these composites was demonstrated in another own work [16], while this paper presents the influence of the direction of the mixture compaction on selected parameters of composites, such as thermal conductivity, capillary rise, as well as flexural and compressive strengths. Additionally, a simulation of temperature distribution in various variants of external walls constructed using these composites was performed in the THERM 7.4 program.

## 2. Materials and Methods

### 2.1. Materials and Recipes

**Figure 1:**
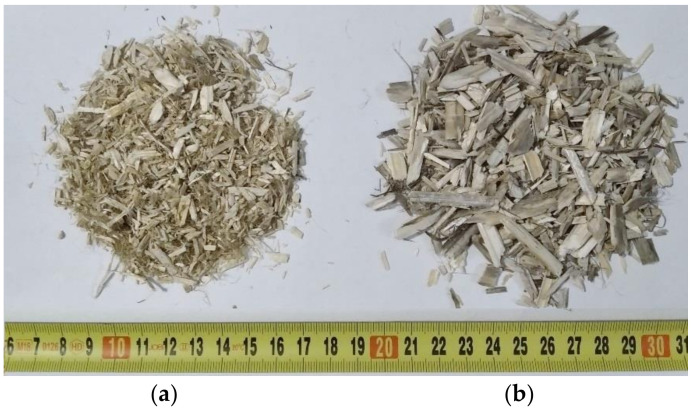
Hemp shives used in the investigation: fine shives (**a**) and thick shives (**b**).

Two mixtures of hemp-lime composite were prepared, differing in the size of hemp shives. The binder used was a mixture of CL–90s hydrated lime (80% by weight) and metakaolin (20% by weight). Pozzolanic materials are usually used in lime and cement to improve the mechanical parameters as well as increase frost and water resistance, in addition to providing a stronger initial bond [24,25,26]. Metakaolin was used as a binder component in hemp-based composites in many studies [27,28,29]. In order to retain water in the binder, and to ensure proper binding process of the components, methylcellulose (0.5% admixture based on the weight of the mixture of lime and methylcellulose) was used. This binder was also used in other studies [27,28]. The weight ratio of shives to binder was taken as 1:2. The weight ratio of water to shives was assumed to be 2.65 for the recipe containing fine shives and 2.9 for the recipe containing coarse shives. Two types of shives were used. Some of them, obtained from the Netherlands (Hempflax), have a smaller fraction, while others—thicker and longer ones—were obtained from a Polish producer (Podlaskie Konopie). The shives used are shown in Figure 1. The average length of the shives is 2.7 mm and 8.4 mm; the maximum length for a representative sample of shives is 11.8 mm (FHS—composite containing fine shives) and 47.4 mm (THS—composite containing thick shives) [16]. The described recipes (Table 1) were also used in another own research [16].

The composites were marked with the symbols THS and FHS, and the description of these symbols is included in Table 2.

### 2.2. Preparation of the Specimens

**Figure 2:**
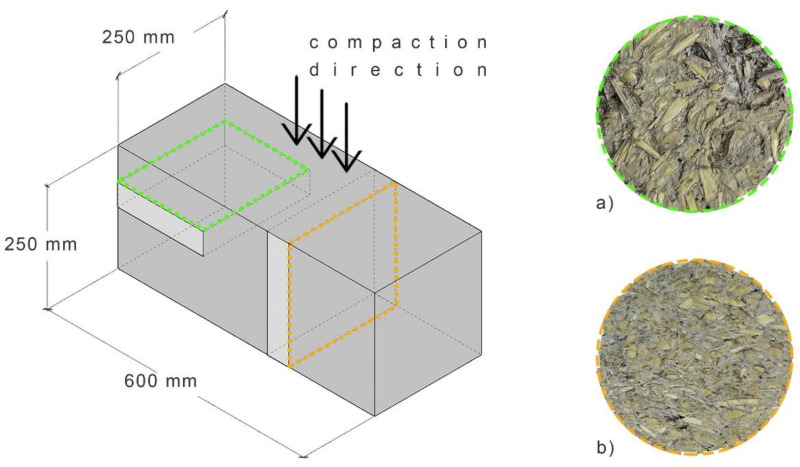
The method of cutting out samples for testing the thermal conductivity coefficient, (**a**) THS ⊥ (**b**) THS II.

**Figure 3:**
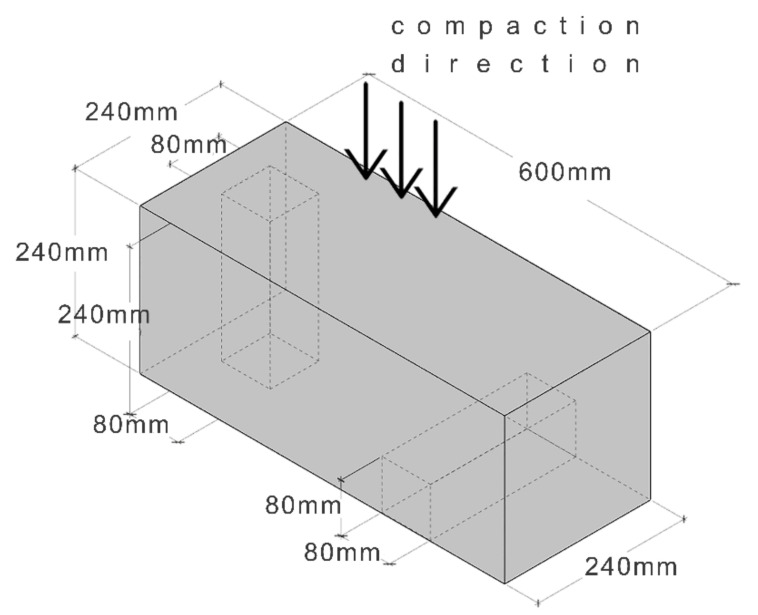
The method of cutting out samples for testing the capillary uptake.

The hemp-lime mixture was arranged in the form of a block with dimensions of 250 mm × 250 mm × 600 mm. The process of laying and compacting the mixture was as follows: First, the mixture was poured into the mold and evenly distributed to a thickness of about 50 mm, then the mixture was compacted by hand in the direction perpendicular to the surface of the layer of the mixture, using a wooden compactor with a cross-section of 30 mm × 30 mm. The tests were performed on the samples cut from a block, after 90 days of curing under laboratory conditions. Two sets of samples with the same dimensions were prepared, but cut in two directions: perpendicular and parallel to the direction of the mixture compaction. In this way, samples with different shives orientation were obtained and it was possible to test the selected properties in two directions: perpendicular and parallel to the compaction direction. The method of cutting out samples is shown in Figure 2 and Figure 3. The dimensions of the cut samples for the thermal conductivity test are 50 mm × 250 mm × 250 mm, while for the capillary uptake test, they are 80 mm × 80 mm × 240 mm. The specimens for the flexural and compressive strength tests were not cut out of the block, but formed in the molds with dimensions of 100 mm × 100 mm × 500 mm (bending) and 150 mm × 150 mm × 150 mm (compression).

The purpose of cutting out samples in two perpendicular directions was to investigate the anisotropy of the composite used as filling of external walls. Manual compaction of the composite causes the shives to fall in the direction perpendicular to compaction. The arrangement of the shives in a fixed direction may affect the different behavior of the material under the influence of external factors acting perpendicularly and parallel to the wall surface.

**Figure 4:**
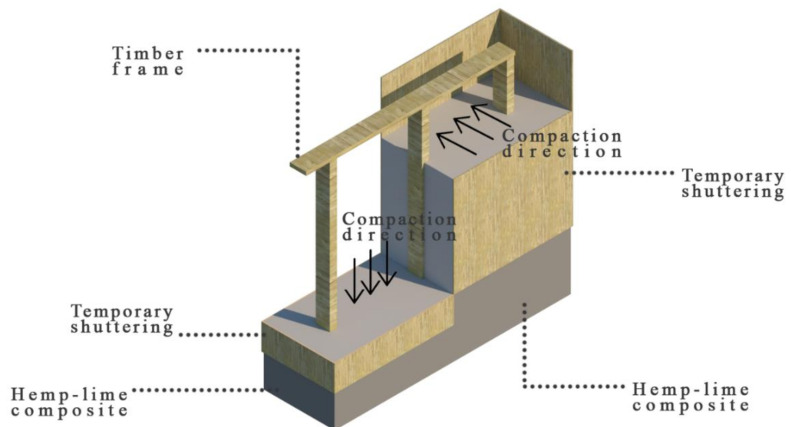
Direction of compacting the hemp-lime mixtures in the timber frame wall.

Usually, the hemp-lime mixture in wall formwork is laid from above, compacting it vertically. Then, the shives run horizontally in the wall. However, there are places in the frame construction where vertical compaction is impossible, and the mixture is laid from the side and compacted horizontally. In these cases, the shives tend to fall horizontally and vertically (perpendicular to the side compaction direction). The described cases are illustrated in Figure 4.

### 2.3. Capillary Uptake

The capillary uptake test was carried out in line with the PN-EN 1925 standard [30]. The employed test methodology was adopted from the standard. Conversely, the sample dimensions as well as the time intervals for weight gain readout were established by the authors. The above-mentioned standard, following adjustment for hemp-lime composite tests, was employed in other studies as well [16,29]. Three 80 mm × 80 mm × 240 mm samples of all mixtures (two sets differ in the direction of water rising in relation to the direction of compaction) were submerged in water to a depth of about 10 mm. The increases in sample mass were recorded in the established time intervals. Thus, it was possible to determine the amount of absorbed water. The sample mass increase was measured after the following time intervals: 15 min, 30 min, 1 h, 3 h, 6 h, 12 h, 24 h, 3 d, 5 d, and 7 d.

### 2.4. Thermal Conductivity

Thermal conductivity was tested on three 250 mm × 250 mm × 50 mm samples of the considered mixtures (two sets differ in the direction of heat flow in relation to the direction of compaction). This parameter was investigated on the basis of the ISO 8302 international standard [31]. Laser Comp Fox 314 (TA Instruments, New Castle, DE, USA) was used in the tests, which were performed with the heat flow meter method. Prior to the test, the samples were subjected to drying in a furnace at 60 °C until constant mass was achieved. In the course of the thermal conductivity test, the hot plate was set at a temperature of 25 °C, whereas the cooling plate was set at 0 °C; thus, the mean temperature obtained amounted to 12.5 °C. The FOX 314 apparatus was characterized by absolute thermal conductivity measurement accuracy of ±2%.

### 2.5. Flexural and Compressive Strengths

The flexural strength test was performed on three 100 mm × 100 mm × 500 mm specimens for each mixture, by means of an MTS 809 hydraulic press 809 (MTS System Corporation, Eden Prairie, MN, USA). Load was applied to the samples via centrally placed force (3-point bending test). The supports were spaced 300 mm apart. The press head displacements amounted to 0.5 mm/min.

In turn, the compressive strength test was conducted on 3 cubic samples per mixture, by means of an MTS 809 hydraulic press (MTS System Corporation, Eden Prairie, MN, USA). The edge length of the samples equaled 150 mm. Since there are no standards for this material, the hydraulic press was set on the basis of arbitrary assumptions. The press head displacements amounted to 0.5 mm/min. The loads and displacements were measured in the course of testing.

### 2.6. Thermal Analysis

Temperature distribution modeling was performed using THERM 7.4 software [32]. It can be employed for two-dimensional modeling of determined heat flow in building elements, partitions, and construction junctions [33,34,35].

In the literature, other partitions made of hemp-lime composite were also analyzed with this software [36]. FEM was employed for solving the heat flow equations. Modeling of junctions involves the following steps [37]:

- model definition (materials, geometry, and boundary conditions),
- mesh generation,
- use of Finite Element Analysis Solver for determining the temperature in nodes and heat streams,
- post-processing and reporting the obtained analytical results (e.g., average heat transfer coefficient for a given node) and the processed results in a graphical form (e.g., isotherms).

The obtained results were used for determining the linear heat transfer coefficient ψ [W/(m∙K)] of a junction in line with the ISO 10211 [38] standard, using the following Formula (1):

(1) $$\psi =L^{2D}-\sum_{i=1}^{j}U_{i}\cdot l_{i}$$

where **L^2*D*^—linear thermal coupling coefficient obtained via a numerical analysis as a product of the average heat transfer coefficient of a junction and its length [W/(m∙K)]; *U_i_* —the heat transfer coefficient of the *i*-th component of the junction [W/(m^2^∙K)], and *l_i_*—the length assigned to the component with the heat transfer coefficient *U_i_* [m].

The U-factor was generated by means of the THERM software. Its values were approximated to those obtained from the calculations for the components of heterogeneous heat layers, according to the ISO 6946 [39] standard.

The thermal properties of individual materials (on the basis of the manufacturer’s data: www.tierrafino.com (accessed on 23 July 2021) [40] and ISO 10456 [41] standard) are summarized in Table 3. In turn, Table 4 presents the boundary conditions employed in modeling, in which the average external temperature for January in Lublin (Poland) was assumed.

#### Analyzed Partitions

**Figure 5:**
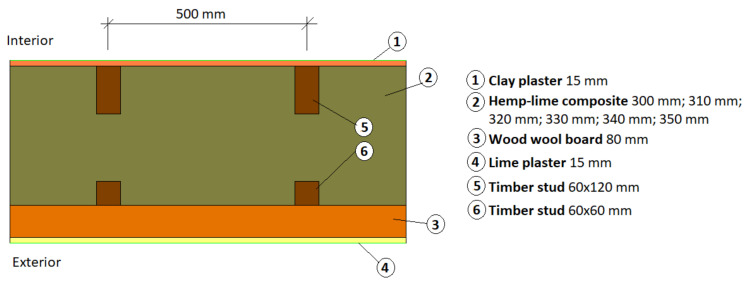
Model of external wall adopted in the simulation.

The analyzed partitions are external walls made of a timber frame structure filled with THS and FHS hemp-lime composite, arranged in two directions: parallel and perpendicular to the wall surface. The thickness of the composite layer was assumed to be 300 mm. In order to meet the current thermal requirements for external walls in Poland, additional wood wool insulation was used from the outside in the form of 80 mm Steico protect panels [42]. The interior is finished with clay plaster, and the exterior with lime plaster. The adopted partition models are shown in Figure 5.

## 3. Results and Discussion

### 3.1. General Characteristics of Composites

In another own work [16], the basic physical properties of the composites analyzed in this article were investigated, such as density, porosity, mass water absorption, specific heat, and vapor permeability. The results are presented in Table 5.

### 3.2. Capillary Uptake

**Figure 6:**
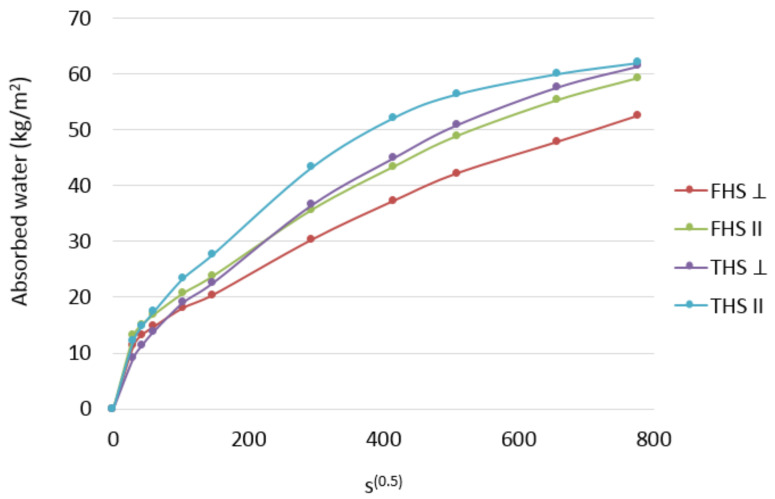
Capillary uptake of the tested composites.

The present results of the capillary uptake phenomenon are shown in Figure 6.

Cutting the samples in a direction parallel to the compaction (THS II and FHS II) and testing these samples in this direction resulted in the shives being directed parallel to the direction of water rising, so the water was pulled across the fibers. On the other hand, cutting out the samples in the direction perpendicular to the compaction (THS ⊥ and FHS ⊥) and testing these samples in this direction resulted in the shives being directed perpendicular and parallel to the direction of water rising, so the water was pulled in the direction across and along the fibers.

The test results indicate a beneficial effect of laying the mixture in the direction of water capillary rise. This dependence applies to the composites prepared according to both recipes.

The composites with longer shives have a greater capillary uptake ability. The most dynamic increase in the amount of absorbed water occurs in the composite containing longer shives, compacted perpendicular to the direction of water rising (THS ⊥). In the case of this composite, the pull-up phenomenon is clearly stabilized and slows down after the fifth day of the test. In the remaining composites, the increase in the absorbed amount of water is approximately constant with time and no slowdown is noticed.

The relationship presented in the diagram shows the influence of shives orientation in relation to the water flow direction. Compaction of the mixture perpendicular to the direction of water uptake is less favorable. Some of the shives are directed with fibers along the direction of the water being taken up. The shives are characterized by the content of pores arranged along the fibers, which contributed to the increase in the amount of absorbed water.

The water absorption coefficients indicate the dynamics of capillary uptake over time. In the literature [27], for similar composites (the same ratio of binder to shives), it was calculated after 24 h of water absorption and ranged from 0.044 to 0.056 kg/(m^2^ s^1/2^). For comparison, the tested composites showed a coefficient in the range of 0.103–0.147 kg/(m^2^ s^1/2^); however, the cited results concerned the composites with a higher bulk density. The calculated coefficients in the case of the water rising parallel to the compaction direction were 17.6% (FHS) and 18.4% (THS) higher than the coefficients calculated in the case of water rising in the direction perpendicular to the compaction direction.

### 3.3. Thermal Conductivity

**Figure 7:**
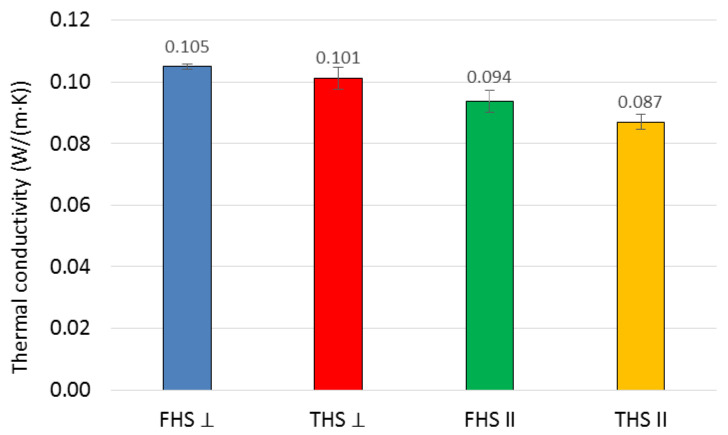
Results of the thermal conductivity test.

The results of the thermal conductivity coefficient tests are shown in Figure 7. Error bars represent the standard deviation.

The direction of the sample cutting with respect to the compaction direction determines the position of the shives. The orientation of the fibers towards the heat flux has an influence on the heat conduction. The materials in which the fibers are directed perpendicular to the heat flow are better insulators. For wood, e.g., pine, two values of the thermal conductivity coefficient are given in the EN ISO 10456 [41] standard—one for heat flow along the fibers, the other across the fibers. The difference in values is significant, reaching as much as 27%. Another example involves the insulation materials made of straw. Straw stalks are thin-walled tubes. The fibers run along the stem. Insulating straw cubes, with the stalks arranged vertically in the wall, i.e., perpendicular to the heat flow, have a lower heat conduction coefficient than the cubes with stalks arranged horizontally, parallel to the heat flow. The literature shows [23] that the straw cubes with stalks arranged perpendicularly to the direction of heat flow are characterized by a 20% lower value of the thermal conductivity coefficient than the straw cubes with stalks arranged parallel to the direction of heat flow.

The results of measurements related to the thermal conductivity coefficient of composites show a clear influence of the compaction direction of the mixture in relation to the heat flow direction. Compaction parallel to the heat flow has proven to be more advantageous. Hemp shives then arranges its fibers perpendicular to the direction of heat flow. The effect is analogous to that in the cited literature. This relationship is more pronounced in the case of a composite containing longer shives. The difference in the lambda value is about 16%, while in the case of composites with fine shives, it is about 12%. The longer the shives, the more susceptible they are to laying perpendicular to the compaction direction; therefore, the effect is more noticeable. In turn, the finer the shives, the greater the probability that they will arrange themselves randomly in relation to the compaction direction. Despite the fact that the FHS composite has a slightly higher porosity, it nevertheless shows a higher thermal conductivity than THS. This may be due to the fact that the finer shives require more binders to achieve the desired consistency, resulting in an increased bulk density, which affects the value of the thermal conductivity coefficient. Williams [1] also showed that the thermal conductivity of the samples tested perpendicular to the direction of compaction is 16% higher than that of the samples tested in the parallel direction. Similar results are also described in [43,44].

### 3.4. Flexural and Compressive Strengths

**Figure 8:**
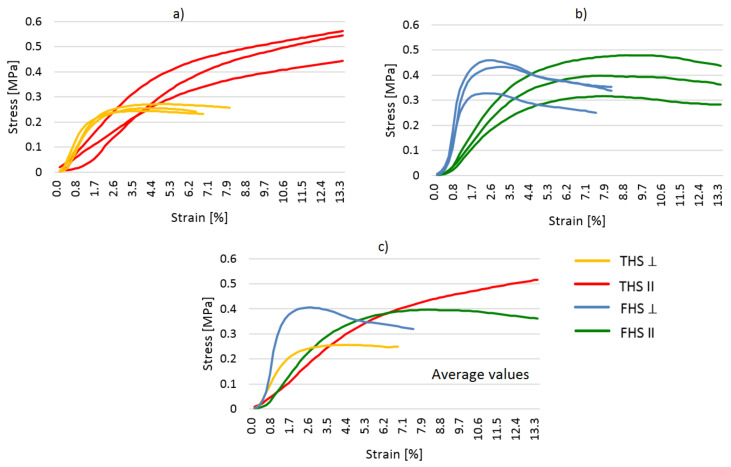
Stress-strain relationship in the compressive strength test, (**a**) THS composites, (**b**) FHS composites, (**c**) Average values (THS and FHS composites).

The results of compressive strength test are shown in Figure 8. Compressive and bending force was applied to the samples in the direction perpendicular and parallel to the direction of compaction. On the basis of the diagrams showing the dependence of force on displacement, it can be assessed that the direction of force application in relation to the direction of compaction has a significant impact on the behavior of the samples under increasing load and on the final strength. Similar observations are described in [19].

The behavior of the samples compressed in two perpendicular directions differs significantly, both in the case of the THS and FHS composites.

In both composites compressed in the direction perpendicular to the compaction of the mixture, the destructive force occurred with a much lower deformation of the samples (THS: 1.7% and FHS: 2.0%). The composites compressed in this direction showed greater stiffness.

In the case of a composite containing fine shives (FHS), regardless of the direction in which the compressive force was applied, the curves followed a similar trend, with a clear peaks of failure (maximum destructive forces). The breaking force was comparable regardless of the direction of the load. Fine shives laid flat, perpendicular to the direction of compaction, provided the composites with greater flexibility, the destruction took place at a significant deformation (approx. 9%).

In the case of shives with longer fractions (THS composites), the behavior of the samples under load is completely different depending on the direction of the applied load. A load parallel to the compaction direction causes the sample to consolidate. In the first phase of loading, the sample is compressed and the binder determines its strength. In this phase, the deformation is small and the stress increase is significant. In the next phase, the binder ceases to fulfill its strength function. The lain shives are compressed and free spaces (voids) between them are eliminated. The deformation progresses quickly and the stress increases more slowly. The angle of the graph becomes smaller. The same observations were described in [20,45,46]. It is not possible to read the material breaking stress. The same problem was noticed by Williams et al. [1]. In the literature, the maximum stress is read at the point of transition from the elastic to plastic state [28,45].

Scientists [47] investigated the effect of the direction of force against the compaction direction on the compressive strength of clay. Researchers showed that the anisotropic properties decreased with increasing compaction force of the clay samples. The compressive strength of baked clay cubes in a direction perpendicular to the casting layers was found to be about 86% of that obtained in a direction parallel to the casting layers.

**Figure 9:**
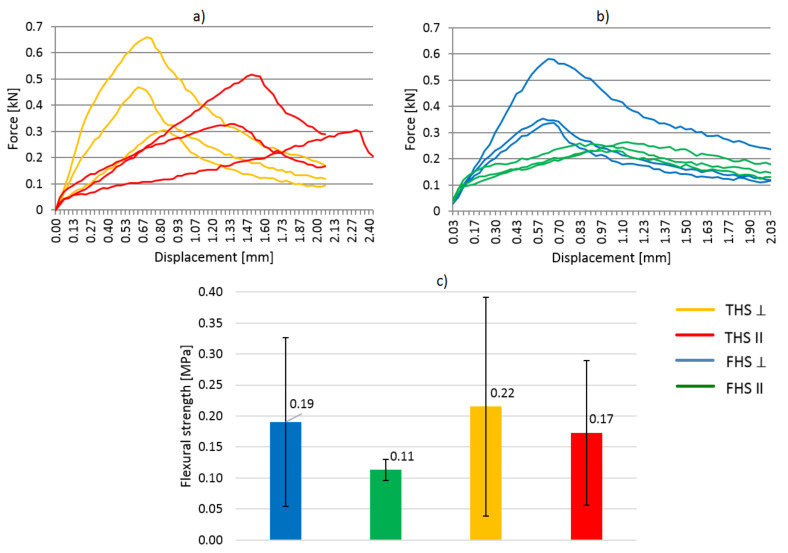
Results of flexural strength test, (**a**) THS composites, (**b**) FHS composites, (**c**) values of flexural strength (THS and FHS composites).

The results of flexural strength test are shown in Figure 9. The behavior of the material under the influence of bending force is quite divergent, as evidenced by significant values of the standard deviation, except for the FHS II formulation. In the case of this recipe, as well as FHS ⊥, the fine shives ensured a more homogeneous distribution of the shives and their better matching, which resulted in a smaller scatter in the results and greater repeatability. Long shives in the THS composite ensured lower homogeneity of the structure and more difficult compaction of the mixture, which resulted in random empty spaces between the shives. Despite the large scatter in the results, the samples of THS composites showed greater strength. Longer shives acted as reinforcement. Comparable strength values are presented in [29], where the composites with the same weight ratio of shives to binder were tested.

The samples loaded perpendicular to the compaction direction (THS and FHS) showed greater stiffness because the failure occurred at a deflection of about 0.5 mm (THS) and 0.6 mm (FHS). The destruction of the samples loaded parallel to the compaction direction took place with a much greater deformation of the samples.

### 3.5. Thermal Analysis

**Figure 10:**
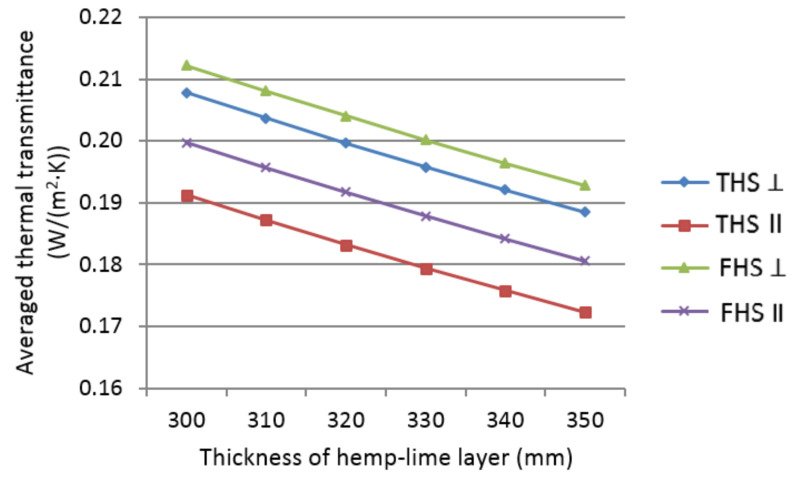
Averaged thermal transmittance coefficient of walls insulated with the analyzed composites, depending on the thickness of the hemp-lime composite layer.

The graph below (Figure 10) presents the changes in the value of the averaged thermal transmittance coefficient of walls insulated with the analyzed composites, depending on the thickness of the hemp-lime composite layer.

The considered external walls are characterized by the mean U-factor value from 0.176 to 0.212 W/(m^2^·K), which is reduced as the thickness of the hemp-lime composite layer increases. The changes in the averaged thermal transmittance coefficient value are rectilinear in relation to the thickness of the composite layer. The effect of the fact that the thinner the composite layer, the greater the share of the timber surface in the design cross-section of the wall is imperceptible in the results.

Referring to the value of the averaged thermal transmittance coefficient (Figure 10), in the case of THS, compacting in the direction of heat flow reduces the coefficient value by about 0.017 W/m^2^ K compared to compacting in the direction perpendicular to the heat flow. In the case of FHS, the difference is lower and amounts to approx. 0.012 W/m^2^ K in favor of compacting in the direction of heat flow. This allows for the reduction of the wall thickness and the reduction of material costs. The wall made of FHS II (compacted in the direction of heat flow) with a thickness of 300 mm meets the Polish technical regulations WT21 [48], i.e., the criterion of 0.2 W/m^2^ K, and in the case of a FHS ⊥ (compacted perpendicular to the heat flow), it must be 330 mm. A composite based on longer shives (THS), thermally more advantageous than FHS, compacted in the direction of heat flow, at a thickness of less than 300 mm meets the Polish technical regulations WT21, and a wall insulated with THS ⊥ (compacted perpendicular to the heat flow) must be 320 mm to meet them. The direction of compaction (shives orientation) as well as the fraction of shives affects the thermal parameters of the wall.

**Figure 11:**
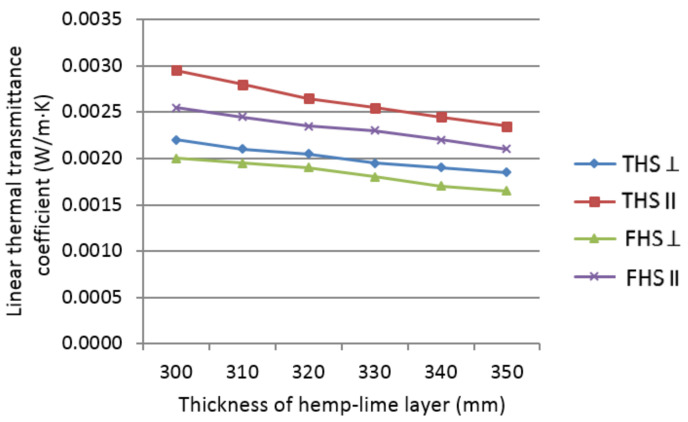
Linear thermal transmittance coefficient of walls insulated with the analyzed composites, depending on the thickness of the hemp-lime composite layer.

The graph in Figure 11 presents the changes in the value of the linear thermal transmittance coefficient characterizing walls of different timber elements, depending on the axial spacing of timber studs.

Timber studs create linear thermal bridges, expressed as linear thermal transmittance coefficient *ψ* ranging from 0.0017 to 0.0029 W/(m·K). This coefficient decreases along with increasing wall thickness in all analyzed cases. The decrease of lambda value of the composite negatively affects the local thermal parameters of the area near the timber frame compared to the filler area, which can be observed as thermal bridges. The changes in the value of the thermal bridge are linear with the changes in the thickness of the composite layer.

## 4. Conclusions

The article presents the studies of the anisotropic properties of the hemp-lime composite prepared according to two recipes, differing in the fraction of hemp shives. Additionally, a simulation of temperature distribution in the walls made with the use of these composites was performed.

A thorough analysis of the obtained results allows drawing the following conclusions:

- The direction of heat flow in relation to the direction of compaction of the hemp-lime mixture in the formwork affects the thermal conductivity of the composite. The difference in the lambda value in the case of THS is about 16%, while in the case of the composites with fine shives (FHS), it is about 12%. The value of the thermal conductivity coefficient is lower in the case of the heat flow in the direction parallel to compaction.
- The direction of compaction of the mixture influences the course of the capillary uptake of water by the composite. A smaller amount of water pulled up by capillary action occurred in the case of the samples compacted in the direction parallel to water uptake. The difference in the water absorption coefficient in the case of THS is about 18.4%, while in the case of the composites with fine shives (FHS), it is about 17.6%. The composites containing longer and thicker shives (THS) showed a greater tendency towards the water uptake by capillary action.
- The direction in which the compressive force is applied has a significant impact on the strength of the composite and its behavior under increasing load. Loading the samples in the direction of mixing the mixture results in greater deformability with a slower increase in stress. On the other hand, the samples loaded in the direction perpendicular to compaction are stiffer. The destruction in this case is more pronounced and occurs with less deformation. The material behavior when the force direction is changed is more varied in the case of the THS composite.
- The direction of bending of composite samples in relation to compaction of the mixture influences its flexural strength and deformability under loading. Both composites (THS and FHS) showed greater stiffness and flexural strength, being loaded perpendicular to the direction of mixture compaction. The difference in the flexural strength in the case of THS is about 29%, while in the case of the composites with fine shives (FHS), it is about 73%. The composites containing longer and thicker shives (THS) are characterized by greater strength, and—at the same time—greater capacity for deformation.
- The thermal simulation of the partition showed that the timber studs create the linear thermal bridges in the wall insulated with a hemp-lime composite. The linear thermal transmittance coefficient decreases together with the increasing layer thickness of each of the tested composites. In a wall insulated with a composite having the lowest thermal conductivity (THS ⊥), the value of this coefficient is the highest. By compacting the mixture parallel to the direction of heat flow, it is possible to reduce the thickness of the hemp-lime insulation layer by 30 mm in order to meet the Polish thermal requirements (U < 0.2 W/m^2^ K), compared to composites compacted in the direction perpendicular to the heat flow.

## Figures and Tables

**Table 1:** Components of composites in kg per 1 m^3^ of the mixture.

Components	THS	FHS
Hydrated lime	205.8	213.4
Metakaolinite	51.4	53.4
Fine hemp shives	-	133.4
Thick hemp shives	128.6	-
Methylcellulose	1.28	1.33
Water	442.4	434.9

**Table 2:** Parameters of the analyzed materials.

Composite Symbol	Description
FHS II	Composite containing fine shives, samples tested parallel to the compaction direction
THS II	Composite containing thick shives, samples tested parallel to the compaction direction
FHS ⊥	Composite containing fine shives, samples tested perpendicular to the compaction direction
THS ⊥	Composite containing thick shives, samples tested perpendicular to the compaction direction

**Table 3:** Thermal conductivity of building materials and elements.

Building Material/Element	Thermal Conductivity [W/(m∙K)]
Hemp-lime composite	0.087/0.094/0.101/0.105
Clay plaster	0.91
Lime render	0.80
Hemp wool	0.046
Timber construction element	0.16

**Table 4:** Boundary conditions adopted in modeling.

Surface	Temperature [°C]	Surface Resistance [(m^2^·K)/W]	Description
Internal	+20	0.13	Heat flow horizontal, simplified *
External	−2.6	0.04	Simplified *
Cut-off planes	‒	‒	Adiabatic

* The simplified model means that convective and radiative heat transfer is described by one common surface resistance.

**Table 5:** The basic physical properties of the tested composites [16].

Parameter (Unit)	FHS	THS
Apparent density (kg/m^3^)	382.4	376.9
Total porosity (%)	82.2	81.7
Mass water absorptivity (after 7 days) (%)	126.9	115.5
Specific heat (J/(kg∙K))	1575	1601
Water vapor diffusion resistance factor (-)	4.94	5.57

## Data Availability

Data is contained within the article.

## References

[1] Williams J., Lawrence M., Walker P. (2016). The Influence of the Casting Process on the Internal Structure and Physical Properties of Hemp-Lime. Mater. Struct..

[2] Suchorab Z., Brzyski P., Raczkowski A., Garbacz M., Życzyńska A. Laboratory Determination of Hygric and Thermal Anisotropy of Aerated Concrete. Proceedings of the AIP Conference Proceedings.

[3] Malaga-Toboła U., Łapka M., Tabor S., Niesłony A., Findura P. (2019). Influence of Wood Anisotropy on Its Mechanical Properties in Relation to the Scale Effect. Int. Agrophys..

[4] Cabrillac R., Fiorio B., Beaucour A.L., Dumontet H., Ortola S. (2006). Experimental Study of the Mechanical Anisotropy of Aerated Concretes and of the Adjustment Parameters of the Introduced Porosity. Constr. Build. Mater..

[5] Brzyski P., Suchorab Z., Malec-Marczewska A. Laboratory Determination of Hygric and Thermal Anisotropy of Hemp-Lime Composite. Proceedings of the AIP Conference Proceedings.

[6] Fort R., Varas M.J., Alvarez de Buergo M., Martin-Freire D. (2011). Determination of Anisotropy to Enhance the Durability of Natural Stone. J. Geophys. Eng..

[7] Kretschmann D. (2003). Velcro Mechanics in Wood. Nat. Mater..

[8] Cordin M., Bechtold T., Pham T. (2018). Effect of Fibre Orientation on the Mechanical Properties of Polypropylene—Lyocell Composites. Cellulose.

[9] Madhuri K.S., Rao H.R., Reddy B.C.M. (2017). Effect of Fiber Orientation and Loading on the Tensile Properties of Hardwickia Binata Fiber Reinforced Epoxy Composites. Int. J. Pure Appl. Math..

[10] Jackson P.W., Cratchley D. (1966). The Effect of Fibre Orientation on the Tensile Strength of Fibre-Reinforced Metals. J. Mech. Phys. Solids.

[11] Hossain M.R., Islam M.A., Vuurea A.V., Verpoest I. (2013). Effect of fiber orientation on the tensile properties of jute epoxy laminated composite. J. Sci. Res..

[12] Stevulova N., Kidalova L., Junak J., Cigasova J., Terpakova E. (2012). Effect of Hemp Shive Sizes on Mechanical Properties of Lightweight Fibrous Composites. Procedia Eng..

[13] Stevulova N., Kidalova L., Cigasova J., Junak J., Sicakova A., Terpakova E. (2013). Lightweight Composites Containing Hemp Hurds. Procedia Eng..

[14] Bourdot A., Moussa T., Gacoin A., Maalouf C., Vazquez P., Thomachot-Schneider C., Bliard C., Merabtine A., Lachi M., Douzane O. (2017). Laboratory Characterization of a hemp-based agro-material: Influence of starch ratio and hemp shive size on physical, mechanical, and hygrothermal properties. Energy Build..

[15] Balčiūnas G., Vėjelis S., Vaitkus S., Kairytė A. (2013). Physical Properties and Structure of Composite Made by Using Hemp Hurds and Different Binding Materials. Procedia Eng..

[16] Brzyski P., Gładecki M., Rumińska M., Pietrak K., Kubiś M., Łapka P. (2020). Influence of Hemp Shives Size on Hygro-Thermal and Mechanical Properties of a Hemp-Lime Composite. Materials.

[17] Nguyen T., Picandet V., Carre P., Lecompte T., Amziane S., Baley C. (2010). Effect of Compaction on Mechanical and Thermal Properties of Hemp Concrete. Eur. J. Environ. Civ. Eng..

[18] Pierre T., Colinart T., Glouannec P. (2014). Measurement of Thermal Properties of Biosourced Building Materials. Int. J. Thermophys..

[19] Nozahic V., Amziane S., Torrent G., Saïdi K., De Baynast H. (2012). Design of Green Concrete Made of Plant-Derived Aggregates and a Pumice-Lime Binder. Cem. Concr. Compos..

[20] Williams J., Lawrence M., Walker P. (2018). The influence of constituents on the properties of the bio-aggregate composite hemp-lime. Constr. Build. Mater..

[21] Cabrillac R., Billoet J.L., Ami-Saada R. (1992). Influence de la porosité et de sa configuration sur le comportement mécanique et thermique des matériaux poreux anisotropes. Cahi. Rhéol.(Paris).

[22] Cabrillac R., Malou Z., Dumontet H. Study of the influence of shape and orientation of the pores on the rigidity of porous materials through a homogenization method. Proceedings of the Sixth International Conference Computers in Composite Materials CADCOMP 98.

[23] Minke G., Mahlke F. (2005). Building with Straw: Design and Technology of a Sustainable Architecture.

[24] Keppert M., Urbanová M., Brus J., Čáchová M., Fořt J., Trník A., Scheinherrová L., Záleská M., Černý R. (2017). Rational Design of Cement Composites Containing Pozzolanic Additions. Constr. Build. Mater..

[25] Toutanji H.A., TaharEl-Korchi T. (1995). The influence of silica fume on the compressive strength of cement paste and mortar. Cem. Concr. Res..

[26] Gameiroa A., Santos Silva A., Faria P., Grilo J., Branco T., Veiga R., Velosa A. (2014). Physical and chemical assessment of lime-metakaolin mortars: Influence of binder:aggregate ratio. Cem. Concr. Compos..

[27] Walker R., Pavía S. (2014). Moisture Transfer and Thermal Properties of Hemp-Lime Concretes. Constr. Build. Mater..

[28] Walker R., Pavia S., Mitchell R. (2014). Mechanical Properties and Durability of Hemp-Lime Concretes. Constr. Build. Mater..

[29] Brzyski P., Suchorab Z. (2020). Capillary Uptake Monitoring in Lime-Hemp-Perlite Composite Using the Time Domain Reflectometry Sensing Technique for Moisture Detection in Building Composites. Materials.

[30] Polish Committee for Standardization (1999). Natural stone test methods. Determination of Water Absorption Coefficient by Capillarity.

[31] European Committee for Standardization (1991). Thermal Insulation—Determination of Steady-State Thermal Resistance and Related Properties—Guarded Hot Plate Apparatus.

[32] Windows & Daylightning, Building Technology & Urban Systems. https://windows.lbl.gov/.

[33] Real S., Gomes M.G., Moret Rodrigues A., Bogas J.A. (2016). Contribution of Structural Lightweight Aggregate Concrete to the Reduction of Thermal Bridging Effect in Buildings. Constr. Build. Mater..

[34] Murad C., Doshi H., Ramakrishnan R. (2015). Impact of Insulated Concrete Curb on Concrete Balcony Slab. Procedia Eng..

[35] Stazi F., Tomassoni E., Bonfigli C., Di Perna C. (2014). Energy, comfort and environmental assessment of different building envelope techniques in a Mediterranean climate with a hot dry summer. Appl. Energy.

[36] Grudzińska M., Brzyski P. (2019). The Occurrence of Thermal Bridges in Hemp-Lime Construction Junctions. Period. Polytech. Civ. Eng..

[37] Mitchell R., Kohler C., Zhu L., Arasteh D., Carmody J., Huizenga C., Curcija D. (2011). Therm 6.3/Window 6.3 NFRC Simulation Manual.

[38] European Committee for Standardization (2017). Thermal Bridges in Building Construction. Heat Flows and Surface Temperatures. Detailed Calculations.

[39] European Committee for Standardization (2017). Building Components and Building Elements. Thermal Resistance and Thermal Transmittance. Calculation Methods.

[40] Tierrafino. http://www.tierrafino.com/.

[41] European Committee for Standardization (2007). Building Materials and Products. Hygrothermal Properties. Tabulated Design Values and Procedures for Determining Declared and Design Thermal Values.

[42] STEICO Engineered by Nature. https://web.steico.com/en/.

[43] Amziane S., Nozahic V., Sonebi M. (2015). Design of Mechanically Enhanced Concrete Using Hemp Shiv. Acad. J. Civ. Eng..

[44] Dinh T.M., Magniont C., Coutand M., Escadeillas G. (2015). Hemp Concrete Using Innovative Pozzolanic Binder. Acad. J. Civ. Eng..

[45] Elfordy S., Lucas F., Tancret F., Scudeller Y., Goudet L. (2008). Mechanical and Thermal Properties of Lime and Hemp Concrete (“Hempcrete”) Manufactured by a Projection Process. Constr. Build. Mater..

[46] Bevan R., Wooley T. (2008). Hemp Lime Construction—A Guide to Building with Hemp Lime Composites.

[47] Zardari M.A., Lakho N.A. (2018). Effect of Compaction during Casting on Anisotropic Compressive Strength of Baked Clay. Teh. Vjesn..

[48] Rozporządzenie Ministra Infrastruktury w Sprawie Warunków Technicznych, Jakim Powinny Odpowiadać Budynki i ich Usytuowanie. http://isap.sejm.gov.pl/.

